# Nimesulide-induced Erythema Multiforme Major: A Case Report

**DOI:** 10.31729/jnma.8762

**Published:** 2024-09-30

**Authors:** Agnimshwor Dahal, Prakash Dhungel, Astha Thapa, Jeevan Gyawali, Amin Shah, Pujan Khadgi, Bhim Chauhan, Prashanna Dip Karki, Nistha Shahi

**Affiliations:** 1 Nepal Intensive Care Research Foundation, Kathmandu, Nepal; 2 Patan Academy of Health Sciences - School of Medicine (PAHS-SOM), Lalitpur, Nepal; 3 Shahid Dharmabhakta Transplant Center, Bhaktpur, Nepal; 4 Nepal Medical College, Kathmandu, 44600, Nepal; 5 National Trauma Center, Kathmandu, Nepal; 6 Karnali Province Hospital, Surkhet, Nepal

**Keywords:** *drug reaction*, *erythema multiforme*, *hypersensitivity*, *knowledge*, *nimesulide*

## Abstract

Erythema Multiforme Major (EMM) is a rare hypersensitivity reaction that primarily affects the skin and mucosa, typically self-limiting. This case discusses a patient who developed severe EMM lesions after consuming nimesulide, a non-steroidal anti-inflammatory drug (NSAID), for abdominal discomfort. Patient was admitted to the hospital, the drug was discontinued, and supportive measures such as pain management and fluid replacement were initiated. This case, being the first to identify nimesulide as a possible trigger for EMM, aims to contribute to the growing body of knowledge on this topic. It emphasizes the need for vigilance and swift intervention for unusual drug reactions.

## INTRODUCTION

Erythema Multiforme (EM), a rare hypersensitivity reaction, affecting the skin and mucosa, predominantly occurs in young adults, with a higher incidence in males.^1,2^

EM is linked to infections, certain drugs, malignancies, and collagen vascular diseases.^3^ It is categorized into Erythema Multiforme minor (EMm) and major (EMM), with EMM involving more widespread lesions and mucosal involvement in 2-10% of cases.^2^ Nimesulide, a COX-2 inhibiting NSAID, has raised concerns due to hepatotoxicity.^4^

## CASE REPORT

A 21-year-old male with no co-morbidity presented to the emergency with abdominal pain for five days with skin rash and fever. The abdominal pain was gradual, on the right side, and continuous. The patient started developing pruritic skin rash two days after taking nimesulide 100mg twice a day for abdominal pain, starting from scrotum which generalized to palms, soles, and entire trunk and later progressed to involve lips and oral mucosa. The fever started three days before the presentation.

**Figure 1:**
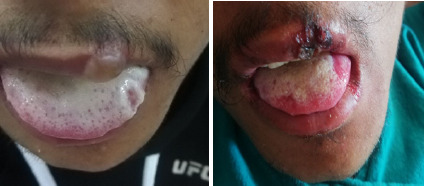
1a, Picture showing bullous lesion on upper lip and white patchy lesion on tongue on day 1; 1b, Picture showing crusted lesion on upper lip and receding white patchy lesion on tongue on day 3

**Figure 2:**
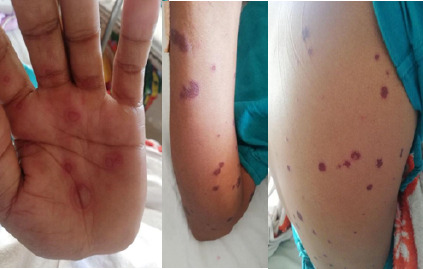
2a, Picture showing erythematous targetoid lesions with an erythematous halo on the palms; 2b, Picture showing erythematous targetoid lesions on the forearm; 2c, Picture showing erythematous targetoid lesions on the back

**Figure 3:**
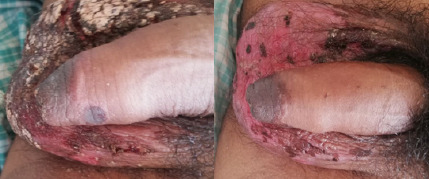
3a, Picture showing ulcerative lesion over the shaft of penis with hyperpigmentation on day 1; 3b, Picture showing hyperpigmented lesions gradually replacing ulcers over the shaft of penis on day 5

On examination, multiple tender ulcers with yellowish-white slough in the buccal mucosa, hard palate, and lateral tongue (Figure 1). Abdominal examination, tenderness with guarding in the right iliac fossa and multiple, discrete, non-tender, target-like papules and plaques with dusky erythematous centre and erythematous margins over the trunk, upper and lower extremities, also involving palms and soles (Figure 2). Genital examination, multiple crusted excoriated patches with central erythema and scab over the scrotum. Ulcerative lesions over the shaft of the penis and vesicular rash at the tip of the penis were present (Figure 3).

The patient's blood reports revealed a hemoglobin of 12 g/dl with a normal total leukocyte count of 6500/cu mm. The chest x-ray of the patient was reported as normal. An abdominal ultrasound revealed echogenic mesentery indicative of inflammatory changes as bowel wall thickening and enlarged lymph nodes in the ascending colon, suggestive of acute colitis. Herpes Simplex Virus (HSV) antigen was negative.

**Figure 4:**
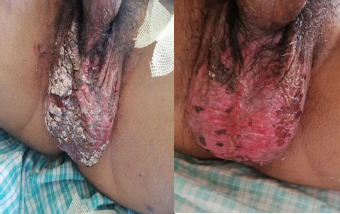
4a, Picture showing erythematous lesions scattered with silvery patch over the lesions and breach of skin on scrotum on Day 1; 4b, Picture showing improving erythematous lesions with some hyperpigmentation on the scrotum on Day 5

The patient was initiated on Ciprofloxacin (200 mg, twice daily) for acute colitis and nimesulide was discontinued. He was managed as an inpatient for a week. He was treated with oral prednisolone at 1mg/kg/day, and an ulcer dressing was performed. The distribution of skin lesions increased until the third day of admission, after which regression was observed (Figures 1, 3, and 4).

He was diagnosed with EMM and acute colitis and was treated with oral steroids, ciprofloxacin, and acyclovir. The patient improved over a week, with the treatment of oral lesions also included, and was discharged with a tapering steroid. During a follow-up visit a week later, lesions had almost resolved, leaving behind some hyperpigmentation.

## DISCUSSION

EM is a hypersensitivity reaction that manifests as skin lesions, typically triggered by certain medications and specific infections. ^1,2^ Our case a 21-year-old male who exhibited a severe variant of this condition, known as EMM, following the consumption of Nimesulide over two days. This patient's demographic profile, being a young adult male, is consistent with the most common demographic for this condition.^2^ The patient presented with macules, papules, vesicles, and bullae, reflecting the "multiforme" nature of EM. There was the presence of the hallmark target lesion, characterized by a red macule with a pale, eroded center.^5^

After ruling out the primary triggers of EM, such as antibiotics like penicillins, cephalosporine and macrolides and infections like herpes, we established a clinical diagnosis of EMM caused by NSAID. A biopsy was conducted, and it revealed vacuolar interface dermatitis with a significant presence of lymphocytes at the dermo-epidermal junction.^1^

EM involves injury to the epithelial cells by cell-mediated immunity. The early phase consists of macrophages and CD8 T cells releasing cytokines and causing inflammation. Oral steroids thin out the immune cells at the site of inflammation, thereby aiding symptoms and recovery.^1,2^

The primary condition that needs to be differentiated from EM is the spectrum of Stevens-Johnson Syndrome/Toxic Epidermal Necrolysis (SJS/TEN). In our case, several factors clearly distinguished the diagnosis. These included the absence of urethritis, lack of widespread bullae formation, the typical age of onset (20-30 years), male gender, presence of a characteristic targetoid lesion with three rings as opposed to the atypical target lesion in SJS/TEN which has two rings, absence of Nikolsky's sign, and the presence of round, red, swollen papules surrounded by areas of skin lightening, contrasting with the flat, red lesions seen in SJS/TEN.^1,2,6^

Mucosal EM can lead to significant discomfort and should be managed with potent topical corticosteroid gels and oral antiseptic.^1,2^ We administered quadragel ointment (a combination of lignocaine, chlorhexidine, and metronidazole) for mucosal lesions. In severe cases, mucocutaneous EM can result in reduced oral intake, necessitating hospitalization for intravenous fluids and electrolyte replenishment.^1^

The most common cause of EM, HSV, was less likely because our patient's urethral discharge resolved without painful genital lesions, which did not fit typical HSV timing.^2^ Also, the temporal relationship between discontinuation of Nimesulide and resolution of EM favors Nimesulide as a cause of EM.

We were unable to find any existing research articles that identify nimesulide as a cause of EM, making this discussion potentially the first of its kind. This highlights the need for further research to gather more evidence. It's crucial to promptly assess skin lesions that present with such widespread involvement and identify the causative agent.

In conclusion, this case underscores the complexity of diagnosing and managing EM, particularly when it presents as a hypersensitivity reaction to medications such as Nimesulide. It's essential to distinguish it from its counterpart, the SJS/TEN spectrum. The patient's demographic, clinical presentation and the temporal relationship between medication use and symptom onset were all factors in reaching a diagnosis. This case serves as a reminder of the importance of considering all potential causes of EM, including less common ones such as Nimesulide, and of the need for a prompt, multifaceted approach, and comprehensive management to ensure the best possible outcomes.
